# Evaluating Patterns of a White-Band Disease (WBD) Outbreak in *Acropora palmata* Using Spatial Analysis: A Comparison of Transect and Colony Clustering

**DOI:** 10.1371/journal.pone.0021830

**Published:** 2011-07-19

**Authors:** Jennifer A. Lentz, Jason K. Blackburn, Andrew J. Curtis

**Affiliations:** 1 Department of Oceanography and Coastal Sciences, Louisiana State University, Baton Rouge, Louisiana, United States of America; 2 Emerging Pathogens Institute and the Department of Geography, University of Florida, Gainesville, Florida, United States of America; 3 Department of American Studies and Ethnicity, University of Southern California, Los Angeles, California, United States of America; King Abdullah University of Science and Technology, Saudi Arabia

## Abstract

**Background:**

Despite being one of the first documented, there is little known of the causative agent or environmental stressors that promote white-band disease (WBD), a major disease of Caribbean *Acropora palmata*. Likewise, there is little known about the spatiality of outbreaks. We examined the spatial patterns of WBD during a 2004 outbreak at Buck Island Reef National Monument in the US Virgin Islands.

**Methodology/Principal Findings:**

Ripley's K statistic was used to measure spatial dependence of WBD across scales. Localized clusters of WBD were identified using the DMAP spatial filtering technique. Statistics were calculated for colony- (number of *A. palmata* colonies with and without WBD within each transect) and transect-level (presence/absence of WBD within transects) data to evaluate differences in spatial patterns at each resolution of coral sampling. The Ripley's K plots suggest WBD does cluster within the study area, and approached statistical significance (*p* = 0.1) at spatial scales of 1100 m or less. Comparisons of DMAP results suggest the transect-level overestimated the prevalence and spatial extent of the outbreak. In contrast, more realistic prevalence estimates and spatial patterns were found by weighting each transect by the number of individual *A. palmata* colonies with and without WBD.

**Conclusions:**

As the search for causation continues, surveillance and proper documentation of the spatial patterns may inform etiology, and at the same time assist reef managers in allocating resources to tracking the disease. Our results indicate that the spatial scale of data collected can drastically affect the calculation of prevalence and spatial distribution of WBD outbreaks. Specifically, we illustrate that higher resolution sampling resulted in more realistic disease estimates. This should assist in selecting appropriate sampling designs for future outbreak investigations. The spatial techniques used here can be used to facilitate other coral disease studies, as well as, improve reef conservation and management.

## Introduction

Over the past three decades, the incidence of coral disease has increased from sparse, localized sightings, to an apparent panzootic, as disease sightings have become commonplace among the world's reef systems. Since the first documented cases of coral disease in the late 1960s and early 1970s [1]–[4], scientists have been working to identify causes of these diseases [5], [6]; however, progress has been slowed by the complexity of coral ecosystems and anthropogenic influences on these systems [5]–[15]. Given the corresponding increase in human population pressure during this time period, it has been suggested that anthropogenic related stressors are contributing to, if not directly causing, coral disease outbreaks [5], [9], [16]–[23]. While correlations between anthropogenic stressors and disease frequencies have been seen for quite some time [15], [17], [24]–[27], it was only recently that direct experimental evidence was able to actually show how anthropogenic stress factors (such as climate change, water pollution, and overfishing) were directly contributing to coral disease [6], [26], [28], [29].

While coral diseases are occurring globally, their incidence appears to be the most severe in the Caribbean [9], [11], [12], [26], [30]–[39]. Over the past few decades reports show that disease is responsible for a roughly 80% loss in Caribbean coral cover [24], [40], [41]. Within the Caribbean, the *Acropora* coral genus appears to have been the hardest hit by disease, with *A. palmata* showing a 90–95% decline [12], [42]–[44] and *A. cervicornis* populations collapsing across the region [41], [42], [45], [46], causing them to be the first corals in history to be listed as “threatened” under the United States Endangered Species Act.

In 1977, shortly after the first documented coral disease, black-band disease (BBD) [1], [2], a second “band” disease was also discovered in the Caribbean [3], [44]. This new white-band disease (WBD) has since been found to occur nearly worldwide in coral-supporting latitudes, ranging from the western Atlantic to the Red Sea, South Pacific, and Arabian Sea [25], [45]. However, to date WBD has only been found to occur in the genus *Acropora* [25]. Despite the well-known phenomenon of WBD, far less is known about its etiology, such as specific pathogen or pathogenic communities (e.g. BBD microbial communities) [47], transmission dynamics or routes of infection [9], [23], [48]–[50].

WBD is visually identified by a white band of tissue separating the living tissue from the dead tissue [3]. The specifics of this disease's appearance are important to note because all too often bleached and predated corals are mistaken for WBD [51]. As the disease band moves, coral tissue is found peeling or sloughing off where the white band is, leaving behind exposed white skeleton [3], [50], [52]. In most cases, the coral skeleton does not remain bare for long, as the void is replaced by rapidly colonizing filamentous algae [52]. This, combined with its rapid rate of spread, as much as 2.06 cm^2^/day, enables WBD to be the only known coral disease able to drastically change the structure and composition of reefs [42].

While BBD has been confirmed to be associated with a community of bacteria [36], this has not been confirmed for WBD [44], [45], [53] or yellow band syndrome (YBS) [54]. However, it is often presumed that WBD is caused by a bacterial infection [2], [3], [44], [55], [56]. To date no pathogen has been isolated in pure culture, nor causation proven [43], [48], [49]. However, the repeated findings of distinct differences between the bacterial communities present in healthy versus diseased tissue has lead recent studies to suggest that bacteria are more than just opportunistic invaders but rather appear to be associated with the disease – if not directly responsible for it [43], [48], [49]. Some studies have proposed that WBD may not be pathogen-induced, but rather a biochemical response to some type of coral trauma, in essence a “shut-down-reaction” [2], [52]. Studies show that the frequency and severity of WBD outbreaks over the past 30 years are unprecedented on a paleontological scale, leading many to speculate that anthropogenic stressors are directly associated with disease, although to date no direct evidence of this reported [24], [42], [44], [52]. The stressors that have been implicated include both regional stressors which are caused by the increasing human population levels coupled with anthropogenically driven climate change, as well as local stressors (such as over fishing, sedimentation, habitat destruction, etc.). However, proving that WBD is linked to any of these stressors is quite difficult without a known pathogen or etiologic agent, if one even exists. Further, if WBD is not pathogen induced, but rather the manifestation of the declining health of corals due to increased stress, then theoretically a diseased state could be brought upon by increases in one stressor (such as a dramatic increase in water temperature) or small to moderate increases in multiple stressors; in which case the stressors involved would likely vary from case to case.

**Figure 1:**
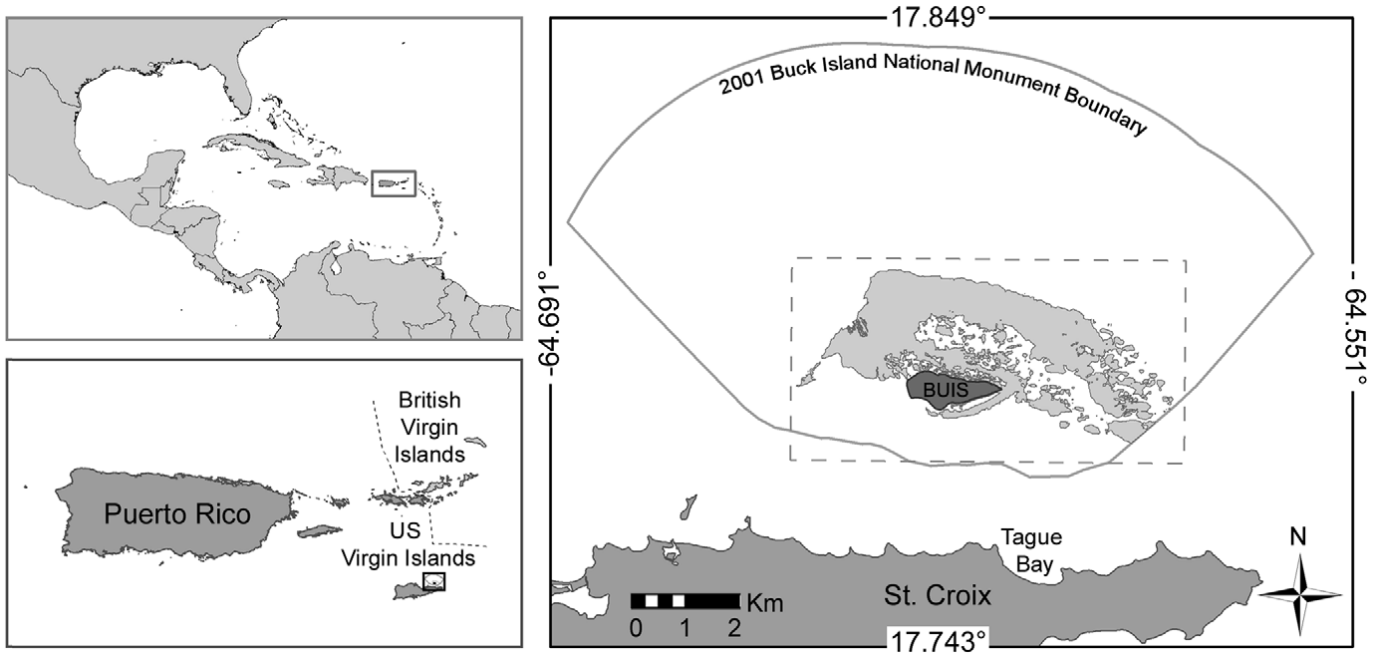
The study area. Buck Island (BUIS) Reef National Monument, located just north of the island of St. Croix, US Virgin Islands (USVI). Mayor et al. 's [56] study area is delineated by the light grey area surrounding BUIS, consisting primarily of hard-bottom substrate less than 10 m deep. The extent of the grid surface used in the DMAP analysis is depicted by the dashed rectangle surrounding Mayor et al. 's [56] study area.

While there is debate over the causes of WBD, as well as the extent and severity of disease-related mortality in *Acropora*, studies increasingly are showing that virtually all areas of the Caribbean are at risk of degradation [24], [42]. By 1982 Tague Bay (see Figure 1), where Gladfelter first identified WBD in 1977, had lost about 50% of its *Acropora* population (both the shallow occurring *A. palmata* and the deeper occurring *A. cevicornis*). Within five years as much as 95% of the original *Acropora* population had died [57]. The decline in *Acropora* populations is of particular importance because the genus is known for developing the reef framework [58], as well as for providing habitat critical to the support of diverse reef fish populations [59] and other organisms that contribute to the productivity and overall health of the reef [42], [44], [60], [61].

Over the last decade there has been increased recognition that geography plays an important role in coral diseases, marked in large part by the growing number of studies that employ geographic information systems (GIS) technologies and spatial statistics [47], [54], [62]; though to date, relatively few studies have directly analyzed the spatial patterns of diseases in reef communities. Jolles et al. [62] provides a key approach to the application of spatial statistics to explore spatial patterns of aspergillosis (a diseased caused by the fungus *Aspergillus syndowii*) in sea fans to test hypotheses of transmission and infection. The study employed the Ripley's K statistic, a global measure of spatial aggregation, to describe the disease patterns in sea fans of various sizes and from multiple sites to determine whether the distribution of diseased sea fans was random, regular, or aggregated with regard to the underlying sea fan population. By doing this they were able to not only quantify the geographic scale of the disease outbreak, but they were also able to test hypotheses regarding the secondary transmission of *A. syndowii*. Their results showed that where disease prevalence was low, the disease appeared to have a random spatial distribution; which might indicate that the disease was being transmitted by terrestrial sources (such as soil runoff or airborne dust). Conversely, they found that where disease prevalence was high there would be a statistically significant spatial aggregation (cluster) of aspergillosis; which would be more indicative of secondary transmission of the disease through either direct contact (sea fan to sea fan, or through a vector such as fish or snails) or through the water column.

More recently, a similar approach was used to study the spatio-temporal patterns of BBD in order to assess possible disease transmission mechanisms [47]. Specifically, they used the Ripley's K statistic in both their spatial and spatio-temporal analyses to infer transmission patterns and to calculate epidemiologic parameters, such as the basic reproductive number (*R_0_*). Their study found that BBD was spatially aggregated (though not to a statistically significant level) and that as the peak disease season was approached the size of these clusters would increase. The temporal nature of their study enabled them to track disease spread throughout their study area. Over the course of their two year study, they found that newly infected corals were often in close proximity to (or even in direct contact with) already infected corals, indicating that BBD was likely being spread through the water column and by direct contact with infected individuals. Ultimately, they reached a similar conclusion as Jolles et al. [62], stating that the presence of disease clusters were the “hallmark signature for the presence of localized transmission dynamics” (page 9 [47]).

The GIS and spatial analytical methods employed by Jolles et al. [62] and Zvuloni et al. [47] facilitated a better understanding of the etiologies of their respective diseases by examining the spatial disease distribution, and testing hypotheses regarding the mode of transmission and infection. However, it is important to note that both of these studies were based on diseases in which the infectious agent has been identified. Unfortunately, this is not the case for most coral diseases. A novel study by Foley et al. [54] used GIS and spatial analysis (specifically the Ripley K function) to study the spatial distribution of YBS in an effort to infer causation from spatial patterns of disease. The results revealed that while the underlying population of susceptible corals (*Montastrea annularis*) appeared to be strongly spatially aggregated, the distribution of *M. annularis* with YBS was less clustered and more regular [54]. Those results were consistent with hypothesized etiologies in which near shore pathogens or toxins were either directly introducing YBS or indirectly leading to YBS by increasing host susceptibility [54]. They postulated that the lack of disease clustering in a population in which the individuals show a strong spatial aggregation, may indicate that the close proximity of the corals may decrease the risk of infection by creating physical barriers which would inhibit the transmission of the disease agent or toxins [54].

Following the rationale of Foley et al. [54], this paper employs spatial statistics in an effort to characterize the patterns of WBD in *A. palmata* colonies from a 2004 outbreak in the reef system around Buck Island National Monument, St. Croix, US Virgin Islands (USVI, see Figure 1) using data from Mayor et al. [56]. In an effort to characterize the prevalence of WBD and the extent of elkhorn coral damage from disease and hurricane damage, Mayor et al. [56] initiated an intensive sampling effort to map and count colonies of *A. palmata.* That initial study documented a prevalence of ∼3% WBD across colonies and suggested that it may still pose a threat to the Buck Island reef community. This study employs the Ripley's K statistic, and a spatial filtering method to identify local spatial clusters of disease and discusses those in the context of possible causative agents or reef trauma that may assist in the ultimate determination of WBD causation.

## Materials and Methods

Spatial analyses were performed on data provided by the US National Park Service. The dataset was originally compiled in a study examining the distribution and abundance of *A. palmata*, and the prevalence of WBD around Buck Island (BUIS) following a 2004 outbreak [56]. In order to facilitate data collection, the original survey evaluated habitats favorable for *A. palmata*, limiting the survey region to hard-bottom areas less than 10 m deep (depicted as the shaded region around BUIS in Figure 1). A total of 617 locations were randomly selected for 25 m by 10 m transect surveys. Of those transects, 375 contained *A. palmata* colonies. Following the original case definition of Mayor et al. [56], “Elkhorn colonies were considered infected with WBD if they had narrow white bands of exposed skeleton, circling completely around the coral branches, bordered on the upper side by live tissue and on the lower side by dead skeleton covered with algae” (page 240). Of those 375 original transects 44 contained evidence of WBD.

**Figure 2:**
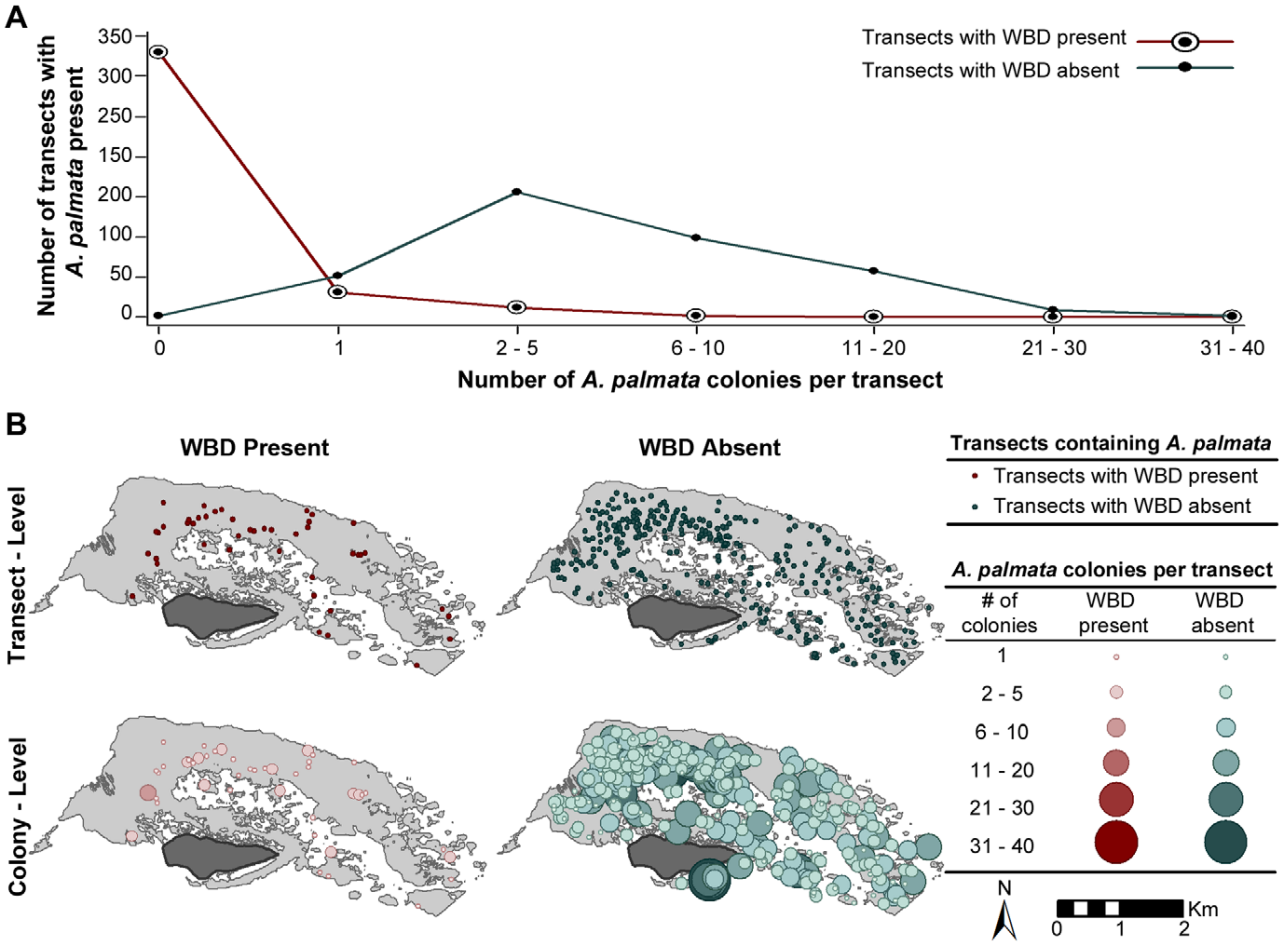
This figure visually depicts the differences between the transect- and colony-level versions of the dataset. (A) Colony densities (the number of colonies per transect) are plotted against the total number of transects with a given colony density, resulting in the cumulative frequency of the colony densities with and without white-band disease (WBD) present. (B) Circular symbols are used to indicate the locations of transects with and without WBD present, from the transect-level version of the dataset (top row). The colony-level dataset is depicted using a graduated symbol map in which the size and color of the symbols used to indicate the locations of each transect are scaled according to the number of colonies within that transect to depicts the colony-level dataset (bottom row).

Spatial locations were recorded for each transect and not for each individual coral colony, though each transect location had a total number of colonies associated with it. To test for potential differences in WBD prevalence estimates and spatial patterns between those two scales, we developed two subsets of spatial data. First, we developed a “transect-level” data set of WBD presence or absence. Second, we developed a colony-level data set that weighted each transect by the number of *A. palmata* and the number of those colonies with WBD (see Figure 2).

### Spatial Autocorrelation Methods

The Ripley's K statistic was employed in ArcGIS 9.3.1 to examine the extent of spatial dependence (the clustering or dispersion of corals) across several distances. This statistic was calculated using the following linear transformation of the K-function: 
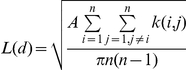
where *n* is either the total number of transects or colonies, *k* is the number of *A. palmata* colonies within the transect, *A* is the study area, and *d* is the distance over which the spatial autocorrelation is being tested. The distance, *d*, was calculated from 0 to 2,500 m in 50 m bins for corals with WBD present, corals without WBD present, and for the underlying coral population for both the transect-level and colony-level subsets. Note no weight was included in the transect-level analyses. A total of six analyses were conducted. For each, 99 permutations were run resulting in a 99% (or 0.01) confidence interval (CI) envelope for the observed Ks. The resultant observed and expected K values (*L(d)* and *d*, respectively) were plotted against the tested distances for each of the 6 analyses. The expected K values represent the null distribution of complete spatial randomness (CSR), also known as the “Poisson distribution.” The plotted expected K values act as the benchmark used to test the spatial distribution of the observed Ks against the null distribution of CSR. The observed Ks that fall along this line are considered to have a spatially random distribution, while anything that lies above this line is considered to have a more aggregated spatial distribution and anything that falls below this line is considered to have a more dispersed spatial distribution. The CI envelope is used to determine whether or not the observed spatial pattern is statistically significant (*p* = 0.01), with no significance associated with the spatial distributions of observed Ks within this envelope. The observed distribution is considered to have significant clustering when the values lie above the upper CI; conversely, values that lie below the lower CI are considered to be significantly dispersed.

**Figure 3 pone-0021830-g003:**
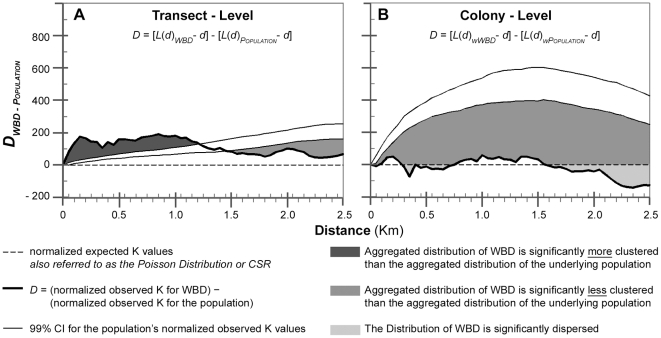
The results of the Ripley's K spatial autocorrelation analysis. Normalized Ripley's K plots were used to assess the spatial distribution of white-band disease (WBD) among *Acropora palmata* over a distance of 2.5 m. Transect-level and colony-level versions of the K function were performed in order to compare the spatial distributions of WBD based on data analyzed at the (A) transect- and (B) colony-levels (respectively). In order to insure that the observed spatial distribution was reflecting the spatial nature of WBD, and not the spatial patterning of the underlying population, the transect and colony-level observed K values for the underlying population were subtracted from the observed Ks of WBD at the transect- and colony-levels, respectively. The resulting K values for WBD were then plotted against distance. The spatial nature of WBD was then assessed by comparing these K values for WBD (thick line) to a spatially random (Poisson) distribution (dashed line at *y* = 0), in which WBD values above the Poisson distribution indicates WBD was aggregated within the underlying population, while values below this line indicated WBD was more dispersed than the underlying population. The 99% confidence intervals (thin lines) generated from the observed K values for the population were used to determine the statistical significance of distribution of WBD within the underlying population of susceptible corals.

We used the difference function (*D*) to examine the spatial distribution of WBD with respect to underlying environmental heterogeneity caused by the presence of the underlying coral population. To do this we subtracted the normalized K values from the underlying population from those of the WBD corals so that we would be able to assess to what extent the spatial distributions of WBD depicted by the homogeneous analyses (see Text S1 and Figures S1–S4) were caused by the disease itself, rather than the natural background variation in the *A. palmata* population (Figure 3). Our resulting Disease-Population difference function was quite similar to the design of the Ripley's K function used by Jolles et al. [62] in which they set their null distribution equal to that of the underlying population of susceptible corals and then plotted *K-K_null_* against distance.

### Spatial Filtering Methods

The Disease Mapping and Analysis Program (DMAP, available for download at http://www.uiowa.edu/~gishlth/DMAP/) was used to employ a spatial filter to smooth prevalence estimates and then identify statistically significant increased prevalence using Monte Carlo simulations [63]–[65]. These prevalence estimates are spatially explicit and represent clusters on the mapped surface. DMAP was used to construct WBD prevalence surfaces for both data subsets.

DMAP analyses require a rectangular gridded surface that encompassed the entire study area. The grid was defined in the northwest by lat/long coordinates of 17.809°N, - 64.648°W, and in the south-east by 17.775°S, - 64.579°E, respectively, with a 50 m^2^ grid cell size (see dashed rectangle in Figure 1). Grid cell size was chosen based on the scale of the analysis and size of the study area. The size of the grid cell is important because it defines the scale of identified cluster patterns, if the grid cells are too small the interpolation will become jagged, while an excessively large grid cell will lack resolution in delineating clusters.

All point level data are aggregated to a filter centered on each grid intersection point. In DMAP this filter is a circle with a user-defined radius. This filter is then applied to the numerator (transects containing WBD positive *A. palmata*) and denominator (all transects containing *A. palmata*) data to calculate prevalence at each grid intersection. It is important to note that these filters must be large enough to cover multiple-grid intersections, allowing for points to be included in multiple prevalence calculations, and thus smoothing the estimated surface which eliminates hard (and often artificially defined) aggregation breaks. Once these local prevalence estimates have been calculated, a Monte Carlo simulation is employed to identify any areas with repeated prevalence estimates higher than expected from the simulations. The Monte Carlo simulation is based on the actual locations of transects containing *A. palmata* colonies; with a probability for each “healthy” individual becoming diseased. Probability was set as the prevalence of each of the transect and colony-level analyses, respectively. A Monte Carlo simulation re-creates this disease surface “*n*” times, creating a simulated distribution against which the actual disease surface is compared. If, for example, the prevalence in one filter is actually higher in 990 out of the 1,000 simulation runs, one can be 99% confident (equivalent to a p-value of 0.01) that the revealed prevalence, or hotspot, did not occur by chance alone. These hotspots are considered spatial clusters of WBD within the BUIS reef system.

As the method of WBD transmission is not currently known, nor the distance to which the pathogen or vector (if any) can viably travel, the spatial parameters used during the spatial analysis could not be based on the epidemiology of WBD. For this reason the optimized bandwidth (*h_opt_*) statistic was used to estimate the size of the spatial filter based on the spatial structure of the dataset. Following Fotheringham et al. [66] the optimized bandwidth was calculated as:
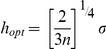
where *n* =  the sample size of transect locations (375) and *σ*  =  the standard distance or a measure of dispersion around the spatial mean of the transect locations. Standard distance was calculated in ArcGIS 9.3.1 using the spatial statistics tool box and a standard deviation of 1 (1688.2 m). The resulting optimized bandwidth estimation (*h_opt_*  = 342.55 m) was employed for DMAP analyses on both transect and colony-level data. Resultant hotspots were mapped in ArcGIS 9.3.1 by rasterizing the DMAP output of the WBD prevalence estimates and overlaying probability value contours outlining disease clusters in which the of WBD prevalence estimates were statistically significant (*p* = 0.05).

## Results

Given that WBD was found at 44 of the 375 transects surveyed, the estimated prevalence of WBD based on the transect-level data was 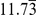
%, suggesting that more than 10% of the transects reported diseased *A. palmata*. However, of the 2,492 colonies surveyed only 69 appeared to have WBD present, which results in a WBD prevalence of 2.77% based on the colony-level data. The mean number of *A. palmata* colonies with WBD absent per transect was 6.48 (min 1, max 40, 5.87 SD), which was very close to that of the overall mean, 6.65 (min 1, max 40, 5.99 SD). While, the mean number of *A. palmata* colonies with WBD present was much lower, 1.57 (min 1, max 6, 1.16 SD). The graph in Figure 2A illustrates the distribution of the number colonies with and without WBD present among the surveyed transects.

As transect- and colony-level analyses were performed on same coral dataset, it became clear how interpretations of the data would change based on the level of reporting (Figure 2B). The transect-level data represent the presence or absence of WBD for each transect, which was visually depicted in the top row of Figure 2B by circles indicating the locations of the 44 transects in which WBD was present (top left) and the 331 transects where no WBD was seen (top right). While, the second version of our dataset, consisted of the same geographic information (the transect locations); it included additional information about the disease-state of the individual colonies within each transect. The colony-level analysis of the dataset was visually depicted by circular-symbols in which the center of each circle indicated the transect location (Figure 2B), while the size and shade of the symbol were scaled to represent the number of colonies within each transect that either had WBD present (bottom left) or WBD absent (bottom right).

The most striking differences between the resultant spatial distributions of the transect- and colony-level versions of the dataset became apparent when the difference function (*D*) was used to examine the spatial patterning of WBD among the *A. palmata* coral populations (Figure 3). The presence/absence analysis of WBD at the transect-level (Figure 3A) revealed spatial aggregation in all transects containing WBD. No significant difference was detected between the aggregated distribution of transects with WBD present and the aggregated distribution of the 375 total transects, based on analysis done using distance thresholds between 1.25 km and 1.50 km; while the aggregation of WBD was found to be significantly more clustered (dark shaded region) at distance scales <1.25 km and significantly less clustered (medium shaded region) at distances >1.50 km than the aggregated distribution of the underlying population. The weighted K function analysis of prevalence WBD at the colony-level (Figure 3B) revealed that colonies with WBD present had fairly random spatial distributions at distances <2.1 km, becoming more dispersed at dispersed at distances >2.1 km. However, when compared to the underlying population densities of susceptible corals, the spatial distribution of the WBD colonies was significantly more dispersed than the aggregated distribution of the susceptible colonies for all tested distances.

**Figure 4:**
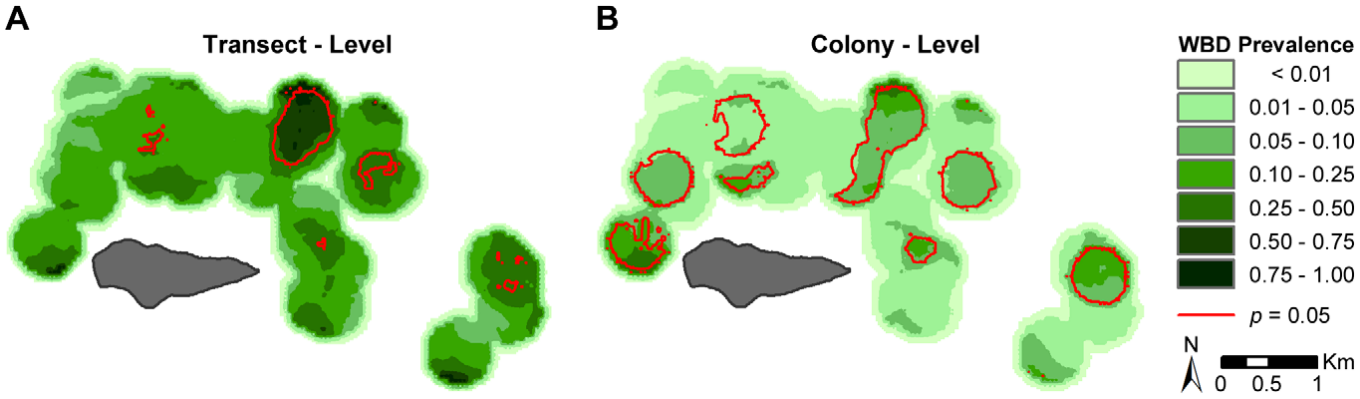
The results of the Disease Mapping and Analysis Program (DMAP) spatial filtering analysis. Comparing the difference between analyzing the coral dataset at the transect (**A**) verses colony-level (**B**) using DMAP. The following spatial parameters were used for both analyses: a 50 m^2^ grid cell resolution; and a 342.55 m filter radius, calculated using the Optimized Bandwidth (*h_opt_*) estimation method. The prevalence of white-band disease (WBD) clustering are shown in green, with darker shades indicating increased prevalence. Areas with statistically significant clustering rates (*p* ≤ 0.05), based on 1000 Monte Carlo simulations, are outlined in red. The numbers placed beside each significant clustering were used solely for identification purposes, and have no empirical value.

Analyses using the DMAP spatial filter revealed significant spatial clustering at both spatial scales tested; however, it is interesting to note some differences in the distribution and size of clusters in each of the two experiments. A red line was used to show the exterior boundaries of areas in which the WBD prevalence estimates were predicted to be statistically significant (*p* = 0.05) based on 1000 Monte Carlo simulations (Figure 4).

Overall, the transect-level analysis revealed relatively high WBD prevalence throughout the study area (indicated by the dark shades of green in Figure 4A), with approximately five areas with statistically significant WBD clustering. By comparing the spatial output to Mayor et al. 's [56] dataset, we found that 36.4% of the transects with WBD present (containing 37.7% of the diseased colonies) were located within 100 m of these five areas of significant disease clustering, with only 13.6% of the WBD transects (containing less than 12% of the total disease colonies) occurring inside one of the areas with significant WBD clustering.

The WBD clustering patterns revealed by the DMAP analysis of the colony-level dataset revealed dramatically different results. The prevalence of WBD was relatively low across the study area, with eight relatively large statistically significant areas of WBD clustering distributed fairly evenly throughout the study area (Figure 4B). When the areas of statistically significant WBD clustering were compared to our underlying dataset, we found that more than half of the transects and colonies with WBD (70.5% and 79.7%, respectively) were within 100 m of one of the 8 significant clustering areas, of which 34.1% of the transects and 50.7% of the colonies were located inside one of the 8 areas.

The total area with significant WBD clustering based on the DMAP Monte Carlo analysis of the colony-level dataset was almost 3 times larger than the total clustering area based on DMAP analysis of the transect-level data (20.50 km^2^ and 7.35 km^2^, respectively), even though the WBD prevalence estimated at the transect level is more than 4 times higher than the prevalence estimated at the colony-level. The mean transect depth inside the significant clustering areas for the transect-level and colony-level datasets was 7.55 m and 6.90 m, respectively, compared to a mean transect depth of 5.87 m for all transects surveyed within the study area.

## Discussion

Despite being one of the first documented coral diseases, there is still little information available on the causative agent or specific environmental stressors that promote white-band disease (WBD) [9], [23], [44], [48]–[50]. As the search for causation continues, surveillance and proper documentation of the spatial patterns may inform etiology, and at the same time assist reef managers in allocating resources to tracking the disease.

Our results show a clear difference between interpreting data at the transect verses colony-level (Figures 2–4). The disease surface produced by the transect-level analysis suggests that this was a severe, widespread WBD outbreak (indicated by the high WBD prevalence estimates throughout the study area, see the dark green areas of Figure 4A). Assuming that the disease is contagious and spreads from an initial location, one could hypothesize that the primary cluster areas identified by the transect-level analysis may be the origin of the outbreak, with cases spreading via the dominant direction of tidal flow, currents, prevailing winds, etc. This hypothesis could be tested with time-specific data on WBD occurrence or modeled with simulated data to determine if such a flow is feasible [47]. This would allow us to develop a working spatial model for contagious spread based on reef morphology, water flow, and environmental conditions around the reef. However, testing this hypothesis was beyond the scope of this study as the dataset we were working with did not have a temporal component. In contrast, the disease surface produced by the colony-level analysis might indicate that a low-grade, broadly distributed WBD outbreak that might be the result of a ubiquitous stressor. In this way, we can use the spatial resolution from each analysis in a Modifiable Area Unit Problem (MAUP [67], [68]) framework and develop field studies and models to test these hypotheses to inform the etiology and subsequent pathogen surveys.

The use of the spatial filtering approach here allowed us to evaluate the distribution of local clusters across the reef and identify specific hotspots of WBD for the 2004 data set. In this way, we can evaluate specific hydrological conditions, reef morphology, or environmental contamination (or microbial communities) that might influence specific regions of the reef that might now be acting globally across reef. While the use of Ripley's K by the seminal works Jolles et al. [62] and Foley et al. [54] provided insights in to the spatial pattern and scale of the apsergillosis in sea fans and YBS in corals, respectively, the precise location of clusters must be inferred in those studies based on sampling strategy and reef location. The Ripley's K statistic is a global measure designed to determine the spatial scale at which clustering is present on the landscape, but it does not identify where on the landscape the clustering is occurring [69]–[72]. As did Jolles et al. [62], Foley et al. [54], and Zvuloni et al. [47], this study directly accounted for the distribution of both infected and unaffected corals, allowing us to test and ultimately reject the hypothesis that clusters in WBD were simply reflections of the underlying coral density. The prevalence of WBD was much lower than the prevalence of aspergillosis in Jolles et al. [62] study in which the mean prevalence among their 3 sites was 47.97%, whereas, the prevalence of WBD was only 2.77% and 11.73% based on the colony- and transect-level datasets respectively. Jolles et al. [62] found significant clustering in areas of high disease prevalence. The Ripley's K results of our transect-level data (Figure 3A) support this, given that the WBD prevalence estimated at the transect-level was much higher than that of the colony-level, and there was the high degree of significant WBD clustering (compared to the aggregated distribution of the underlying transects) based on the transect-level data, whereas no significant WBD clustering was detected using the colony-level Ripley's K analysis of the colony-level data (Figure 3B). However, this does not appear to be the case when the results of the DMAP analyses are examined, as the colony-level data had a total significant clustering area almost 3 times larger than that of the transect-level data, but the WBD prevalence estimated at the transect level is more than 4 times greater than colony prevalence.

The low prevalence of WBD among *A. palmata* colonies, combined with the fairly random spatial distribution of WBD colonies shown in Figure 3B, might indicate that the disease is caused by either air and/or water-born direct transmission of the causative disease agent from a terrestrial point of origin [62]. The rational being that corals “of equal size have equal chances of being hit by infectious material suspended in the water column” (page 2374 [62]). The assumptions of this hypothesized mode of disease transmission were supported given that the overall distance between possible terrestrial-based contaminant sources and the locations of the *A. palmata* colonies is quite large compared to the significantly aggregated spatial distribution among the susceptible colonies [62]. In addition, the dispersed WBD distributions might also indicate that the clustered coral population may offer protection from disease by providing physical barriers to the disease agents or toxins [54].

The presence of statistically significant areas of WBD clustering, as indicated by our DMAP analyses, does not necessarily conflict with the assumptions of this hypothesis, as the type of cluster analysis used to test this theory by the previously mentioned studies (i.e. the Ripley's K function) was based on a global statistic designed to quantify changes in spatial patterns at various distances. Instead, given the low WBD prevalence estimates and broad geographic distribution of the areas with statistically significant disease clusters identified by the DMAP analysis, the colony-level data could be used to support this hypothesis, suggesting that WBD might be the result of a ubiquitous stressor. In such a case, the areas of significant disease clustering, might indicate the presence of locally aggregated stress factors which might make the surrounding corals more vulnerable to infection (suggested by Jolles et al. [62]). This hypothesis could be tested by looking for correlations between areas with increased environmental risk factors and the areas of significant WBD clustering predicted by DMAP (or other types of spatial filtering analysis) in comparison to areas absent of disease in the study area. Conversely, WBD clusters may indicate the presence of diverse microbial organisms with different virulence levels, though the causative agent(s) and mechanism are not yet described.

Disease clustering could also be the result of genetic clustering of corals that are more susceptible to the disease. This possibility was ruled out by both Jolles et al. [62] and Zvuloni et al. [47] as genetic clustering was unlikely due to the reproductive nature of the corals in their studies (sea fans and massive corals respectively). However, while *A. palmata* can reproduce sexually via broadcast spawning (which would make genetic clustering unlikely), their dominant mode of reproduction throughout most of the Caribbean tends to be asexual fragmentation [73]. Historically, *Acropora* relied on seasonal sexual reproduction to increase their population size and distribution, while using asexual fragmentation as a survival mechanism to rebound from storms or other physical damage. Ultimately, one of the traits that had made *A. palmata* so resilient in the past may be a contributing factor to their decline, as the decrease in genetic diversity that tends to occur in populations dominated by fragmentation may cause the corals to be more susceptible to emergent epizootics [74]. In addition, when fragmentation occurs the corals have to devote their energy towards recovery instead of reproduction [73]. The same appears to be true of stress in general for *Acropora,* as populations recovering from disease, bleaching, & other high stress conditions so decreased, or the complete cessation of sexual reproductive processes. It's unclear how long it takes for *A. palmata* to recover from enough from fragmentation or other stresses enough to start spawning. Lirman's [73] study showed that “3 years after Hurricane Andrew, gametes were only present in large *A. palmata* colonies that had not experienced direct fragmentation during the storm. Neither those colonies that were damaged by the hurricane nor any of the hurricane-generated fragments had produced gametes at this time” (page 53). Additionally it appears that “colony fecundity is dependent on a coral's size and condition” (page 124 [75]), which is a problem because stressors appear to disproportionately affect the larger colonies [75].

Overall the combined low disease prevalence, limited number of (large) clusters, and wide distribution of significantly significant WBD clusters suggests WBD may “persist as a ubiquitous, chronic stress,” as was suggested by Grober-Dunsmore et al. [75] for the *A. palmata* in their study area (which surrounded the island of St. John, located just north of Buck Island in the USVI).

At present, many investigations examining spatial data concentrate (and more importantly, collect) only on the variable of interest. In the case of coral disease, this would be the location of the diseased coral. However, without similarly collected denominator data, it is impossible to know if the pattern revealed by the analysis is a disease “hotspot,” or simply indicative of locations with higher densities of coral; i.e., *ceteris paribus*, the more coral there is, the more diseased corals are likely to be found. However, a counter problem of weighting a transect by the number of colonies is – exactly where do colony boundaries occur? It is possible to create an artificial hotspot by adding too many artificial boundaries. For these reasons, studies examining coral diseases should be done at as fine a spatial resolution as possible, with accurate and precise spatial measurements. This will have the added benefit of not only improving existing spatial investigations but opening the analysis to more sophisticated spatial inquiry.

Future studies should also examine each of these significant WBD clustering areas at both the geographic and microbial scales. In this way spatial regression models could be used to associate disease clusters with surrounding environmental factors, such as stressors (human population size, pollution, frequently visited tourist sites, etc.), and/or physical properties (surface currents, sea surface temperatures, wind direction, salinity, etc). Analyses at the microbial scale could test for similarities and differences in the histology and bacterial communities between corals from each of the significant diseased clusters; as well as compare corals within significant disease clustering areas to those in non-significant diseased areas.

The analysis and mapping approach employed here can also be used to study the spatio-temporal changes in coral health by comparing changes in the position, size, and local prevalence rates of clusters and significant areas of coral disease and bleaching. Comparisons of the clustering of different types of diseases present in one location may also provide valuable insight into the continued decline in reef health worldwide. These spatial insights should provide valuable insights to both aspatial coral disease researchers and marine resource managers with information on the most vulnerable areas of the reefs.

## Supporting Information

Text S1 **The Methodology and Results pertaining to Figures S1–S4.**
(DOC)

Figure S1 **Ripley's K plots of the diseased and underlying population at both the transect and colony-levels.** Ripley's K plots comparing the spatial patterning of white-band disease (WBD) and the underlying *Acropora palmata* population, and showing the affect distance has on each of these spatial patterns. The null distribution of complete spatial randomness (CSR) is represented by the Expected K values (*d*) which are equal to the distance interval in which they are being tests (for example, the Expected K value at a distance of 500 m would be 500), thus as the distance threshold increases so will the Expected K values. In all cases the Observed K (thick lines), and their corresponding 99% confidence intervals (thin lines) fell above the CSR benchmark (dashed line) indicating that both WBD and the underlying coral population had aggregated (clustered) spatial distributions across all of the tested distances at both the transect and colony-level. The results of the non-weighted K functions (**A–B**) assess the degree of clustering or dispersion present in the spatial distribution of the transect locations; while the results of the weighted K functions (**C–D**), in which each transect location was weighted by the number of colonies within it, evaluate the degree of clustering or dispersion of the colonies. (**A**) Significant clustering (shaded region) was detected in the spatial distribution of transects with WBD present at distances to ≤1.1 km, and non-significant clustering was detected up to 2.5 km (the maximum distance tested). (**B**) The spatial distribution of the 375 transects containing *A. palmata* showed significant clustering at all of the tested distances. (**C**) When the locations of transects with WBD present were weighted by the number of WBD colonies within them, their resulting spatial distribution was clustered, but not to a statistically significant extent. (**D**) When the transect locations of the underlying population were weighted by the total number of colonies within them, their resulting spatial distribution showed signs of aggregation at all of the distances tested, but only detected significant clustering at distances ≤1.05 km and ≥1.75 km.
(TIF)

Figure S2 **Normalized Ripley's K plots depicting the same information as shown in Figure S1.** The transect locations for both white-band disease (WBD, **A**) and the underlying population (**B**) were clustered at all spatial distances tested (0–2.5 km); with the population showing significant clustering (shaded region) at all distances <2.5 km and significant clustering only occurring at distances ≤1.1 km for transects in which WBD was present. (**C**) Transects containing WBD colonies still appear to be spatially aggregated across all of the tested spatial scales, but not to a statistically significant extent. (**D**) As in the transect-level analysis, the distribution of transects containing both diseased and non-diseased *A. palmata* colonies was also spatially aggregated; however, when the transects are weighted by the number of colonies within them, they only appear to have statistically significant clustering when tested using distances thresholds ≤1.15 or ≥1.7 km.
(TIF)

Figure S3 **Normalized Ripley's K Plots used to test the null hypothesis** ***H_S3_*** **.** Graphical representation of the test of the null hypothesis (*H_S3_*) that transects weighted by the number of colonies within them will not be significantly more clustered or dispersed than the underlying spatial distribution based on the transect locations alone. In order for the null hypothesis to be accepted the observed K based on the colony-level data (thick line) must fall within the upper and lower 99% confidence intervals (CIs, depicted as thin lines) estimated using the transect-level data. (**A**) The null hypothesis was rejected at distances <1.1 km and accepted at distances >1.1 km for white-band disease (WBD). (**B**) The null hypothesis was rejected for the population data at all of the distances tested.
(TIF)

Figure S4 **Normalized Ripley's K Plots used to test the null hypothesis** ***H_S4_*** **.** A graphical representation of the test of the null hypothesis (*H_S4_*) that the spatial distribution of the colony-level data would be more clustered or dispersed than they would be through chance alone. This hypothesis was rejected for both (**A**) white-band disease (WBD) and the (**B**) underlying population because the observed K (thick line) based on the transect-level data falls within the 99% confidence intervals (CIs, depicted as thin lines) based on the observed K estimated using the colony-level data.
(TIF)
