# Supplementary material for: Evaluating Patterns of a White-Band Disease (WBD) Outbreak in Acropora palmata Using Spatial Analysis: A Comparison of Transect and Colony Clustering
Source: PLoS One. 2011 Jul 19;6(7):e21830. doi: 10.1371/journal.pone.0021830 (PMC3139597; doi:10.1371/journal.pone.0021830)
Supplement: Text S1 — The Methodology and Results pertaining to Figures S1–S4. (DOC) [file pone.0021830.s001.doc]

# Supporting Information

# Supplemental Materials and Methods

## Spatial Autocorrelation Methods

The computed Ripley’s K values were plotted against the distances tested in for each of the 6 coral types (Figure S1). To facilitate interpretation of the above spatial distributions, we normalized the Ripley’s K output by subtracting the expected values (*d*) from the observed values (*L(d*)), so our new benchmark for evaluating CSR was *y* = 0 (as opposed to the pre-normalized benchmark of *πd^2^*). In order to determine whether or not clustering was present (and if so whether or not the aggregation was statistically significant) the normalized K values (*L(d) – d*) were then plotted against distance (Figure S2). Next, to test the null hypothesis (*H_S3_*) that transect locations that are weighted by the number of colonies within them are not significantly more clustered (or dispersed) than the underlying pattern of just their locations, we plotted the weighted K using the CIs for the unweighted K (Figure S3). We plotted the reverse combination (unweighted K and the CIs for the weighted K) in order to test the null hypothesis (*H_S4_*) that the colony-level dataset would be more clustered or dispersed than they would be by chance alone (Figure S4).

# Supplemental Results

The Ripley’s K statistics were computed, the underlying coral population continued to show signs of significant clustering throughout all of the tested spatial distances at the transect-level (see Figure S1B and S2B), and for the most of the distances at the colony-level (see Figure S1D and S2D), with the highest degree of significant clustering occurring at distance thresholds of 1450 m and 1700 m for the transect- and colony-level datasets respectively (see Figure S2B,D). Clustering was detected in the distribution of diseased corals for all of the tested distances, with the greatest degree of clustering occurring at distances of 1100 m for the transect-level data (see Figure S2A) and 1400 m for the colony-level data (see Figure S2C), with distances ≤ 1100 m showing statistically significant spatial aggregation of WBD at the transect-level. In both versions of the dataset, the normalized Observed K for WBD takes a sharp dip at distance threshold of 350 m (see circled regions of Figure S2A,C). Overall, the plots based on the normalized Ripley’s K values were preferred over plots based on the raw K values, because when the data was normalized such that the CSR benchmark was set to *y* = 0, the hyperbolic nature of the plots was removed and the resulting graphs were much more expressive.

Figure S3, shows the graphical test of the null hypothesis (*H_S3_*) that transects weighted by the number of colonies within them are not significantly more clustered or dispersed than the underlying spatial distribution based on the transect locations alone. The *H_S3_* hypothesis was rejected for WBD at distances < 1100 m because the colony-level observed K were greater than the upper CI for the transect-level observed K indicating that transects weighted by the number of WBD colonies within them were significantly more clustered than their locations alone would suggest (see Figure S3A). However, *H_S3_* was accepted when WBD was examined at distances > 1100 m, as the observed K for WBD colonies was within the upper and lower CI for the observed K of the transects containing WBD, indicating that the spatial aggregation of WBD was not statistically significant at these distance scales. This hypothesis was rejected for the underlying population for all of the distance scales tested because the colony-level observed K was above the upper CI for the transect-level population data, indicating that the transects weighted by the number of colonies within them were, in fact, significantly more clustered than the spatial distribution of the transect locations alone (see Figure S3B).

Figure S4 shows a graphical test of the null hypothesis (*H_S4_*) that transects weighted by the number of colonies within them would be more clustered or dispersed than they would be by chance alone. *H_S4_* was rejected for both WBD (Figure S4A) and the underlying population (see Figure S4B) because the observed K based on the transect-level data fell within the CI envelope based on the colony-level observed Ks.
