# Supplementary figures and images for: Evaluating Patterns of a White-Band Disease (WBD) Outbreak in Acropora palmata Using Spatial Analysis: A Comparison of Transect and Colony Clustering
Source: PLoS One. 2011 Jul 19;6(7):e21830. doi: 10.1371/journal.pone.0021830 (PMC3139597; doi:10.1371/journal.pone.0021830)

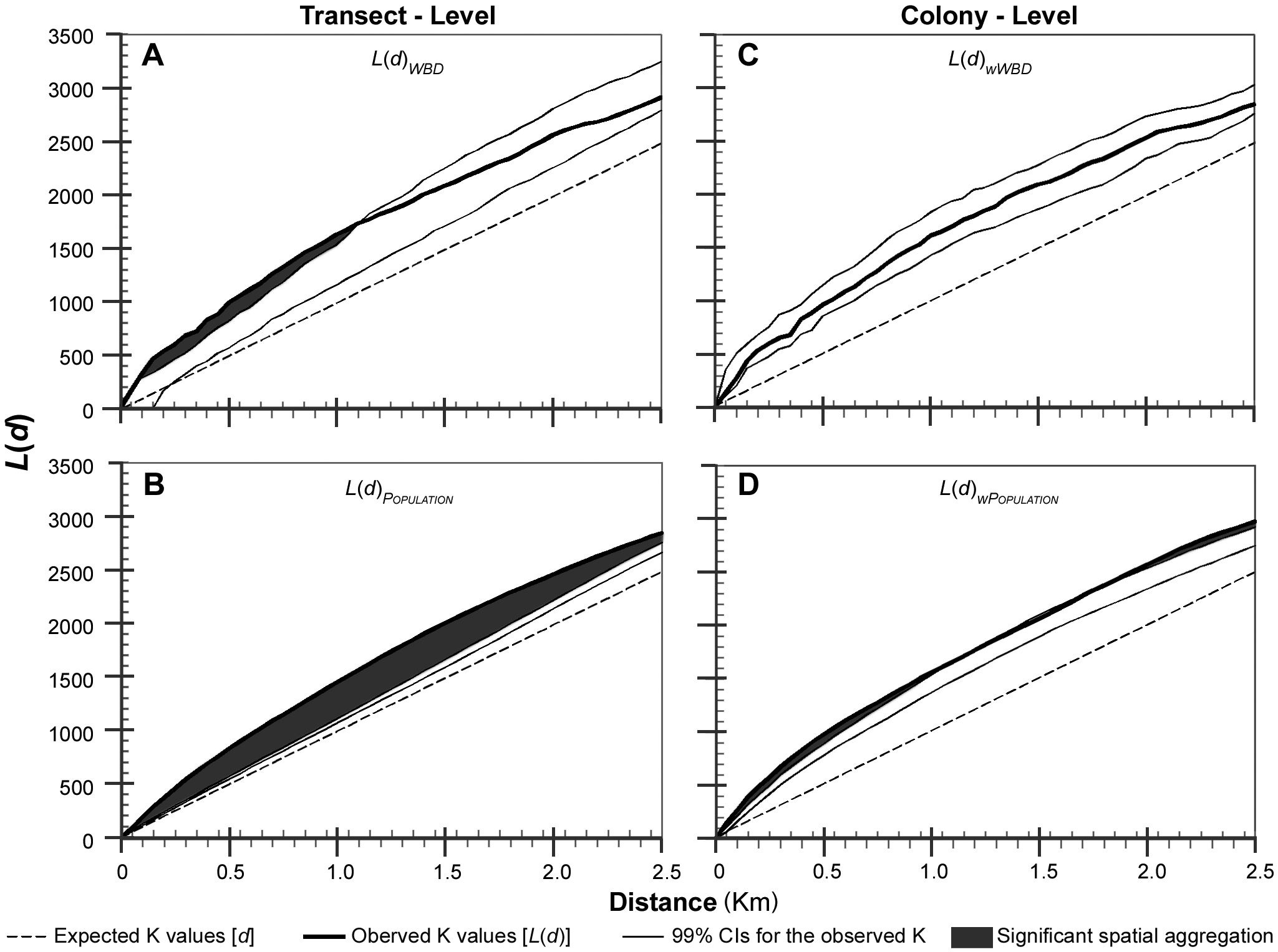

Supplement: Figure S1 — Ripley's K plots of the diseased and underlying population at both the transect and colony-levels. Ripley's K plots comparing the spatial patterning of white-band disease (WBD) and the underlying Acropora palmata population, and showing the affect distance has on each of these spatial patterns. The null distribution of complete spatial randomness (CSR) is represented by the Expected K values (d) which are equal to the distance interval in which they are being tests (for example, the Expected K value at a distance of 500 m would be 500), thus as the distance threshold increases so will the Expected K values. In all cases the Observed K (thick lines), and their corresponding 99% confidence intervals (thin lines) fell above the CSR benchmark (dashed line) indicating that both WBD and the underlying coral population had aggregated (clustered) spatial distributions across all of the tested distances at both the transect and colony-level. The results of the non-weighted K functions (A–B) assess the degree of clustering or dispersion present in the spatial distribution of the transect locations; while the results of the weighted K functions (C–D), in which each transect location was weighted by the number of colonies within it, evaluate the degree of clustering or dispersion of the colonies. (A) Significant clustering (shaded region) was detected in the spatial distribution of transects with WBD present at distances to ≤1.1 km, and non-significant clustering was detected up to 2.5 km (the maximum distance tested). (B) The spatial distribution of the 375 transects containing A. palmata showed significant clustering at all of the tested distances. (C) When the locations of transects with WBD present were weighted by the number of WBD colonies within them, their resulting spatial distribution was clustered, but not to a statistically significant extent. (D) When the transect locations of the underlying population were weighted by the total number of colonies within them, thei [file pone.0021830.s002.tif]

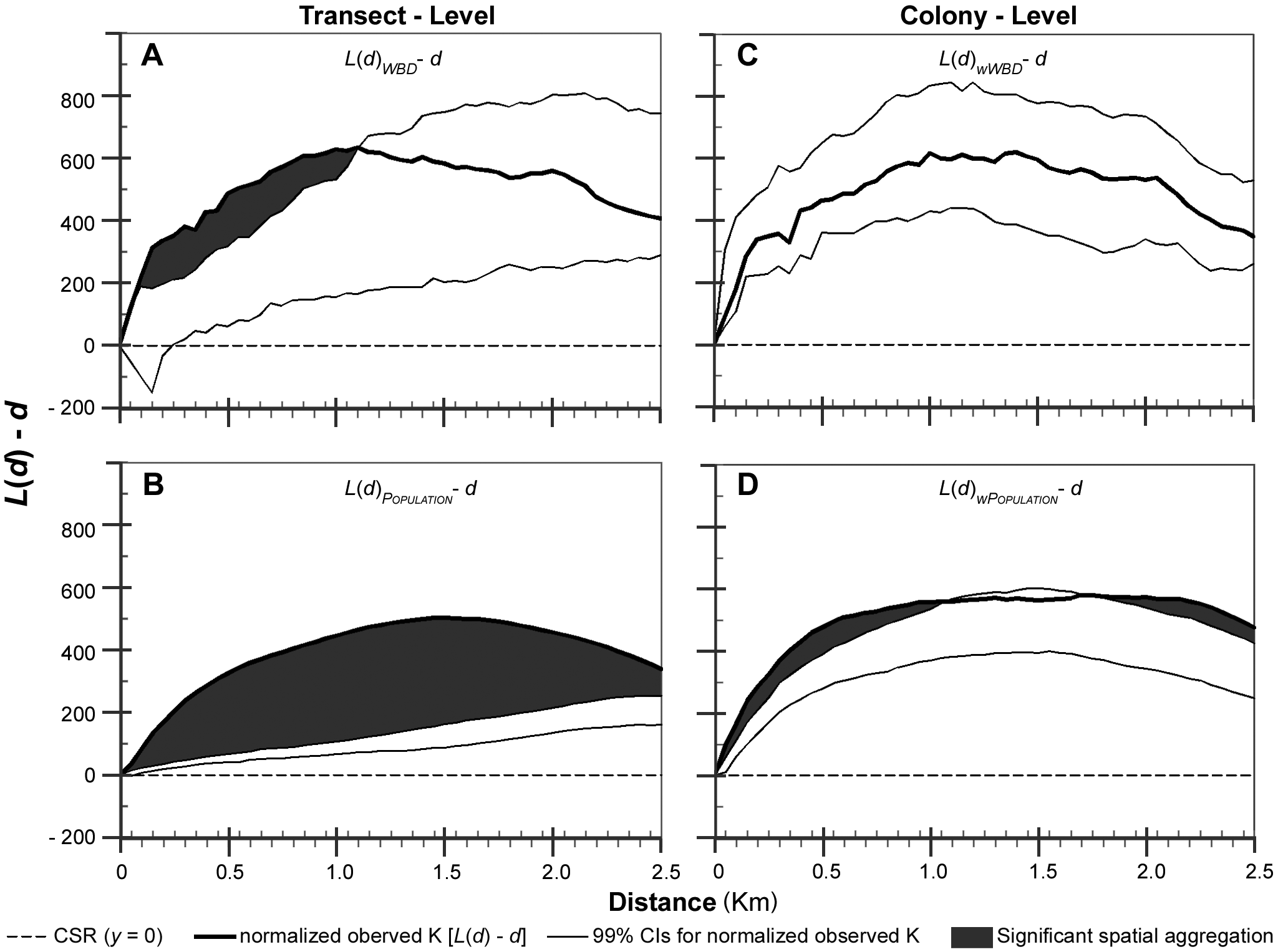

Supplement: Figure S2 — Normalized Ripley's K plots depicting the same information as shown in Figure S1. The transect locations for both white-band disease (WBD, A) and the underlying population (B) were clustered at all spatial distances tested (0–2.5 km); with the population showing significant clustering (shaded region) at all distances <2.5 km and significant clustering only occurring at distances ≤1.1 km for transects in which WBD was present. (C) Transects containing WBD colonies still appear to be spatially aggregated across all of the tested spatial scales, but not to a statistically significant extent. (D) As in the transect-level analysis, the distribution of transects containing both diseased and non-diseased A. palmata colonies was also spatially aggregated; however, when the transects are weighted by the number of colonies within them, they only appear to have statistically significant clustering when tested using distances thresholds ≤1.15 or ≥1.7 km. (TIF) [file pone.0021830.s003.tif]

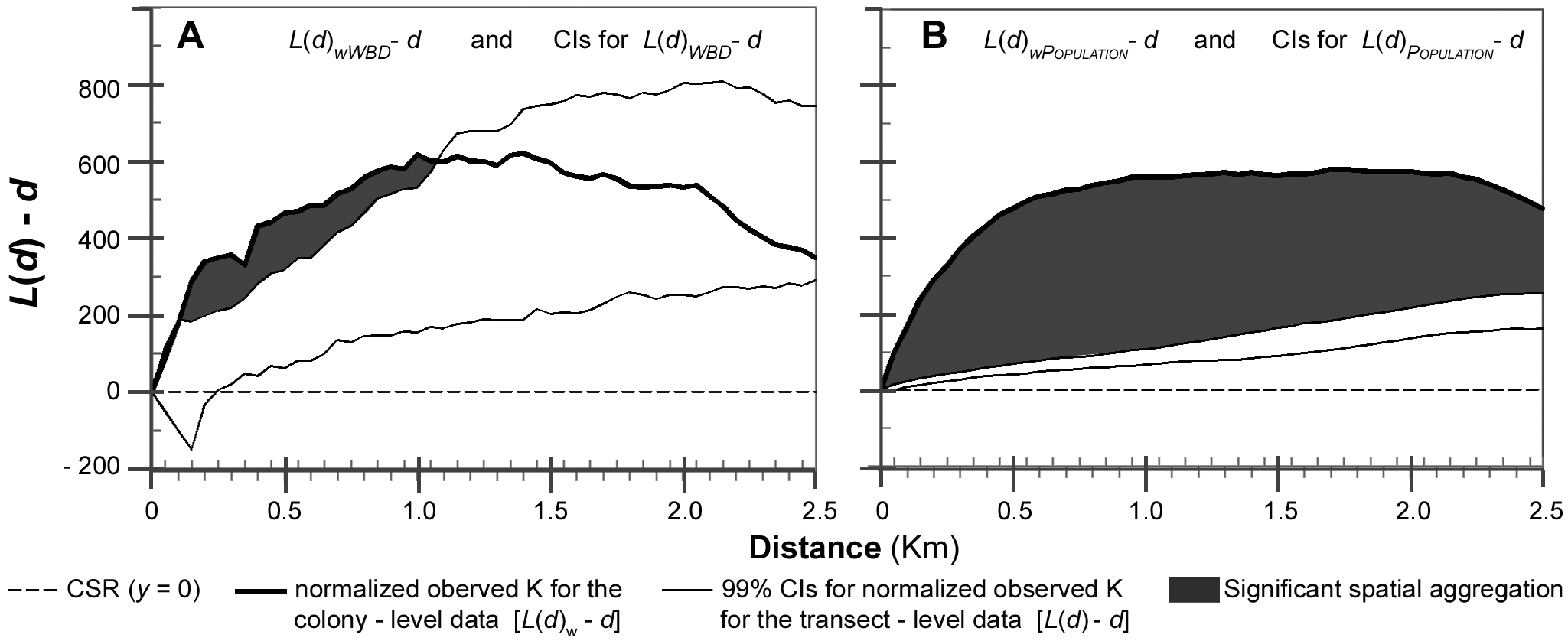

Supplement: Figure S3 — Normalized Ripley's K Plots used to test the null hypothesis HS3 . Graphical representation of the test of the null hypothesis (HS3) that transects weighted by the number of colonies within them will not be significantly more clustered or dispersed than the underlying spatial distribution based on the transect locations alone. In order for the null hypothesis to be accepted the observed K based on the colony-level data (thick line) must fall within the upper and lower 99% confidence intervals (CIs, depicted as thin lines) estimated using the transect-level data. (A) The null hypothesis was rejected at distances <1.1 km and accepted at distances >1.1 km for white-band disease (WBD). (B) The null hypothesis was rejected for the population data at all of the distances tested. (TIF) [file pone.0021830.s004.tif]

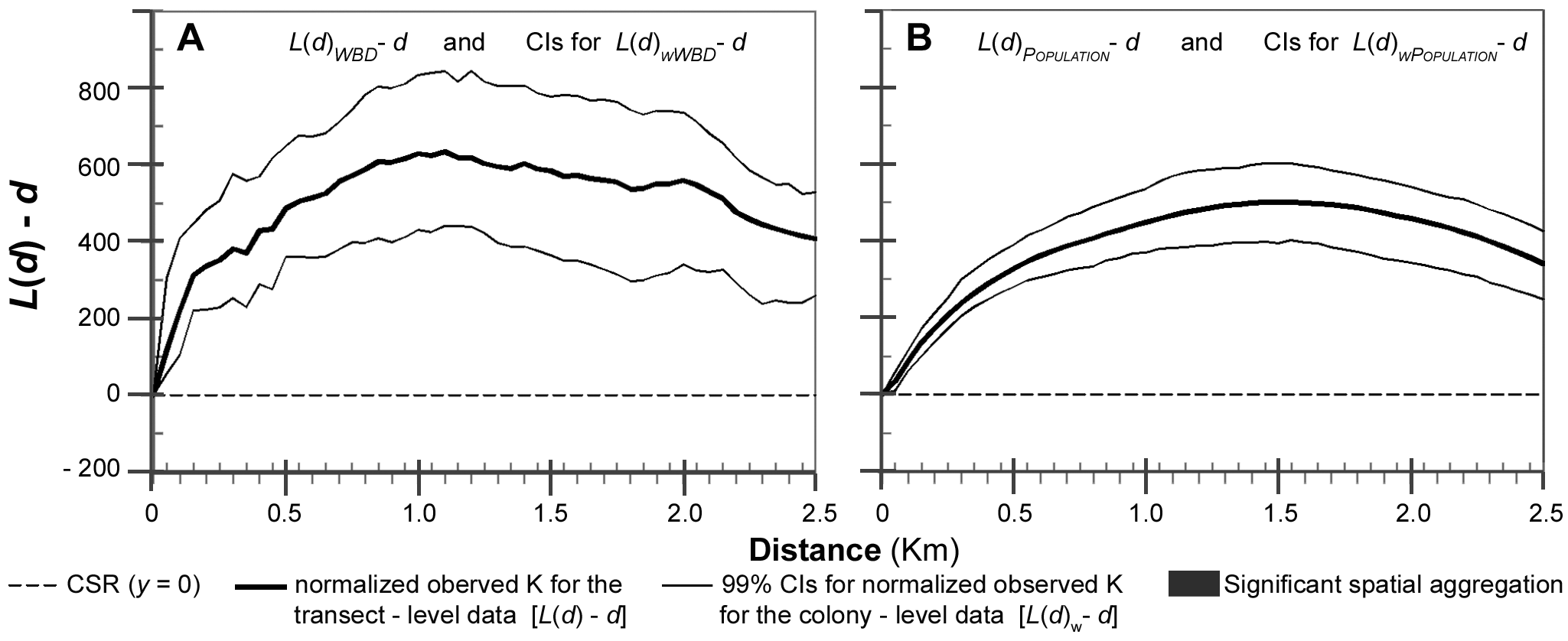

Supplement: Figure S4 — Normalized Ripley's K Plots used to test the null hypothesis HS4 . A graphical representation of the test of the null hypothesis (HS4) that the spatial distribution of the colony-level data would be more clustered or dispersed than they would be through chance alone. This hypothesis was rejected for both (A) white-band disease (WBD) and the (B) underlying population because the observed K (thick line) based on the transect-level data falls within the 99% confidence intervals (CIs, depicted as thin lines) based on the observed K estimated using the colony-level data. (TIF) [file pone.0021830.s005.tif]
